# Parent training for disruptive behavior symptoms in attention deficit hyperactivity disorder: a randomized clinical trial

**DOI:** 10.3389/fpsyg.2024.1293244

**Published:** 2024-02-16

**Authors:** Gabrielle Chequer de Castro Paiva, Jonas Jardim de Paula, Danielle de Souza Costa, Antônio Alvim-Soares, Daniel Augusto Ferreira e Santos, Julia Silva Jales, Marco Aurélio Romano-Silva, Débora Marques de Miranda

**Affiliations:** ^1^Programa de Pós-Graduação em Medicina Molecular da Universidade Federal de Minas Gerais, Belo Horizonte, Brazil; ^2^Faculty of Medicine, Research Center of Impulsivity and Attention, Federal University of Minas Gerais, Belo Horizonte, Brazil; ^3^Departamento de Saúde Mental, Faculdade de Medicina da Universidade Federal de Minas Gerais, Belo Horizonte, Brazil; ^4^Departamento de Pediatria, Faculdade de Medicina da Universidade Federal de Minas Gerais, Belo Horizonte, Brazil

**Keywords:** attention-deficit/hyperactivity disorder, oppositional defiant disorder, ODD, parent training, digital interventions

## Abstract

**Background:**

Attention-Deficit/Hyperactivity Disorder (ADHD) affects 5% of children and 2.5% of adults worldwide. Comorbidities are frequent, and Oppositional Defiant Disorder (ODD) reaches 50%. Family environment is crucial for the severity of behaviors and for prognosis. In middle-income countries, access to treatment is challenging, with more untreated children than those under treatment. Face-to-face behavioral parent training (PT) is a well-established intervention to improve child behavior and parenting.

**Method:**

A clinical trial was designed to compare PT-online and face-to-face effects to a waiting list group. Outcomes were the ADHD and ODD symptoms, parental stress and styles, and quality of life. Families were allocated into three groups: standard treatment (ST), ST + PT online, and ST + Face-to-Face PT. We used repeated measures ANOVA for pre × post treatment analysis corrected for multiple comparisons.

**Results and discussion:**

Parent training was effective in reducing symptoms of ADHD (*p* = 0.030) and ODD (*p* = 0.026) irrespective of modality (*p* = 1.000). The combination of ST and PT was also associated with better quality of life in the physical domain for patients (*p* = 0.009) and their parents (*p* = 0.050). In addition to preliminary data, online intervention seems effective for parenting and improving social acceptance of children. The potential to reach many by an online strategy with a self-directed platform may imply effectiveness with a low cost for public health to support parents’ symptoms management.

## Introduction

Attention-Deficit/Hyperactivity Disorder (ADHD) affects approximately 5% of children and 2.5% of adults worldwide (Posner et al., 2020). The disorder is characterized by symptoms of inattention, hyperactivity, and impulsivity, which are associated with functional impairments in various life domains [American Psychiatric Association (APA), 2014]. ADHD, a heterogeneous condition (Drechsler et al., 2020), significantly impacts school performance, social relationships, and family dynamics (Anastopoulos et al., 2009; Sasser et al., 2017), amplifying academic difficulties, learning impairments, and interpersonal conflicts (DuPaul et al., 2022).

Throughout life, ADHD manifests as self-esteem issues, learning obstacles, disruptive behavior, and socialization during the early school years. In adolescence, it escalates to include defiant behavior, conduct problems, criminal behavior, substance abuse, and school dropout (Harpin, 2005). Consensus in 2021 affirmed the pervasive lifelong impact of ADHD, affecting the quality of life and psychosocial functioning and imposing functional limitations (Faraone et al., 2021), consequently resulting in substantial public costs (Faraone et al., 2021).

Moreover, ADHD often coexists with other psychiatric conditions, amplifying the associated impairments and impacts (Lahey and Willcutt, 2010; Barbaresi et al., 2013). The most common comorbid condition, oppositional defiant disorder (ODD), affects 54–67% of ADHD cases (Do Austerman, 2015). When ADHD combines with ODD, difficult temperament, impulsivity, and challenging behaviors intensify, leading to heightened parental stress and negative parenting tendencies (Modesto-Lowe et al., 2008).

Parents of children with ADHD often resort to less effective parenting strategies due to these challenges. This situation includes increased focus on externalizing problems, repetitive commands, reduced reinforcement, and responsiveness compared to parents of typically developing children (Anastopoulos et al., 2011). The family environment plays a critical role in the severity of behavioral issues, influencing ADHD prognosis and comorbidities (Shaw et al., 2001).

Interventions, particularly parent training, significantly alleviate these symptoms and behaviors, holding promise in minimizing the lifelong impacts of these disorders (Figge et al., 2018). Alongside pharmacological approaches, behavioral interventions, especially parent training, have gained recognition (Murphy et al., 2018; Caye et al., 2019). Parent training exhibits substantial evidence in enhancing the parent–child relationship, increasing parental competence, fostering positive parenting, increasing satisfaction in their parental role and maternal wellbeing, and reducing ODD symptoms (Caye et al., 2019; Kostyrka-Allchorne et al., 2022).

National Institute for Health and Clinical Excellence (NICE) guidelines advocate for behavioral interventions in managing behavioral disorders in children and adolescents (Pilling et al., 2013). These interventions, employing reinforcement-based strategies and social learning principles, aim to enhance desired behaviors and curtail unwanted behaviors, thereby improving conduct (Daley et al., 2018).

However, accessibility to Parental Training (PT) is often limited due to service availability, cost, and logistical challenges. The advent of online PT, proven effective in treating various childhood disorders, presents a promising solution (DuPaul et al., 2014). Research indicates that online PT yields comparable results to face-to-face interventions (Baumel et al., 2016), potentially enhancing treatment accessibility without compromising effectiveness (Baumel et al., 2016, 2021).

Improving parental skills and behavioral control, PT significantly contributes to mitigating ADHD-related challenges (Chronis-Tuscano et al., 2011). Nevertheless, in the Brazilian context, effective public policies for ADHD treatment are lacking, resulting in difficulties for affected children and families to access proper care and school support (Nevison and Zahorodny, 2019). Delays in psychiatric treatment, often up to a decade post-symptom onset, impose substantial costs on emergency health services and school retention. Cost analysis, including emergency health services and school retention costs, concluded that greater investment in psychiatric treatment, as designed in the World Health Organization (WHO) guidelines, would save 3.1 times more considering non-treatment expenses in the 5–19 years (Maia et al., 2016). Access and effectiveness of psychiatric care should be a commitment to promote wellbeing and improve functionality and adaptability (Nevison and Zahorodny, 2019).

Recognizing these challenges and building on existing research, our clinical trial explores a self-directed online behavioral parent training platform in a middle-income controlled scenario. We investigate the effects of PT in two delivery formats: online and face-to-face, regarding ADHD and ODD symptoms, comparing it with standard care.

## Method

### Study design

The PT trial is designed as a randomized, controlled, experimental, open, single center, with three-arm parallel groups. Randomization has been blocked with a 1:1 allocation.

### Ethics and registration

The study was evaluated and approved by the local and the National Ethical Committee; each included family was informed and gave written consent agreeing to participate in the study. The study protocol was registered in the REBEC platform, which is referenced by the clinical trials (UTC number U1111-1293-9285). The study follows the CONSORT principles and statement (Schulz et al., 2015).

This clinical trial was funded by the Coordination for the Improvement of Higher Education Personnel (CAPES); the Research Program for the SUS (PPSUS: Foundation for Research Support of the State of Minas Gerais; Secretary of State for Health of Minas Gerais; Ministry of Health Brazil); and the National Council for Scientific and Technological Development. This funding source had no role in the design of this study and will not have any role during its execution, analyses, interpretation of the data, or decision to submit results. The author(s) declare(s) that they have no competing interests.

### Participants

One hundred and thirty-two children were screened at the Impulsivity and Attention Research Center between March/2021 and May/2022 and were enrolled in our trial after meeting the pre-established inclusion/exclusion criteria. The collection date followed what was proposed in the research project approved by the funding agencies. Fifty-seven children and their families fully participated in the study. Families whose children were boys, aged between 6 and 12 years, and who had externalizing symptoms of hyperactivity/impulsivity and/or defiant behavior participated in the study. Exclusion criteria are as follows: ADHD inattentive without externalizing symptoms; families whose children or caregivers scored below the 5th percentile in standardized IQ tests; children with severe genetic or neurological conditions or with severe psychiatric comorbidities (i.e., psychosis, severe depression, severe autism, bipolar affective disorder); families whose caregivers had a reported severe psychiatric diagnosis (i.e., severe depression, psychosis, and bipolar affective disorder); or have few years of formal education. In addition, families with severe social adversity, such as constant exposure to hunger, violence, or extreme poverty, were also excluded from this research (Kazdin, 2005).

As part of the usual follow-up, families underwent an initial assessment consisting of a clinical interview based on the DSM-5. The interview was conducted by a psychologist from the center with experience and expertise to conduct it. The child and the primary caregiver underwent an intelligence assessment, and the child was also assessed for school performance. As patients were included in the study, permuted blocks of six participants/families were formed, which were randomized 1:1 into three parallel groups stratified by children’s age. Eleven blocks were formed, totaling 66 participants. Of these, 57 concluded their participation in this research, and nine were excluded due to decline in treatment (five; three of GROUP 03 and two of GROUP 02); diagnostic divergence between the screening team and the psychiatric team (one of GROUP 01); recently conducted parent training (one of GROUP 02); duplicate enrollment and screening (one of GROUP 01); and incomplete intervention until completion of data collection (one of GROUP 03). The 1:1 randomization assigned two participants to each of the three groups:

GROUP 01 (*n* = 20): Waiting list for behavioral approach while under standard treatment (ST). Bi-monthly consultations with a child and adolescent psychiatrist, including drug treatment if there is a clinical indication (at medical discretion, according to the clinical protocol), and without any other type of complementary treatment.

GROUP 02 (*n* = 19): Standard treatment and behavioral PT intervention in face-to-face format (ST + Face-to-Face PT). Bi-monthly consultations with a child and adolescent psychiatrist, including drug treatment if there is a clinical indication (at medical discretion, according to the clinical protocol), and complementary behavioral intervention: parent management training, in face-to-face format, with a specialized therapist, in six sessions held on a weekly basis, and adapted from the “Parent Management Training” manual developed by Kazdin (2005).

GROUP 03 (*n* = 18): Standard treatment and behavioral PT intervention in online format (ST + online PT). Bi-monthly consultations with a child and adolescent psychiatrist, including drug treatment if there is a clinical indication (at medical discretion, according to the clinical protocol), and complementary behavioral intervention: parent management training, in an online format, on a platform developed for the study, in six modules, to be carried out on a weekly basis, and adapted from the “Parent Management Training” manual developed by Kazdin (2005).

Concurrent behavioral interventions were not allowed. All families provided consent and assent forms.

### Procedures

**Figure 1:**
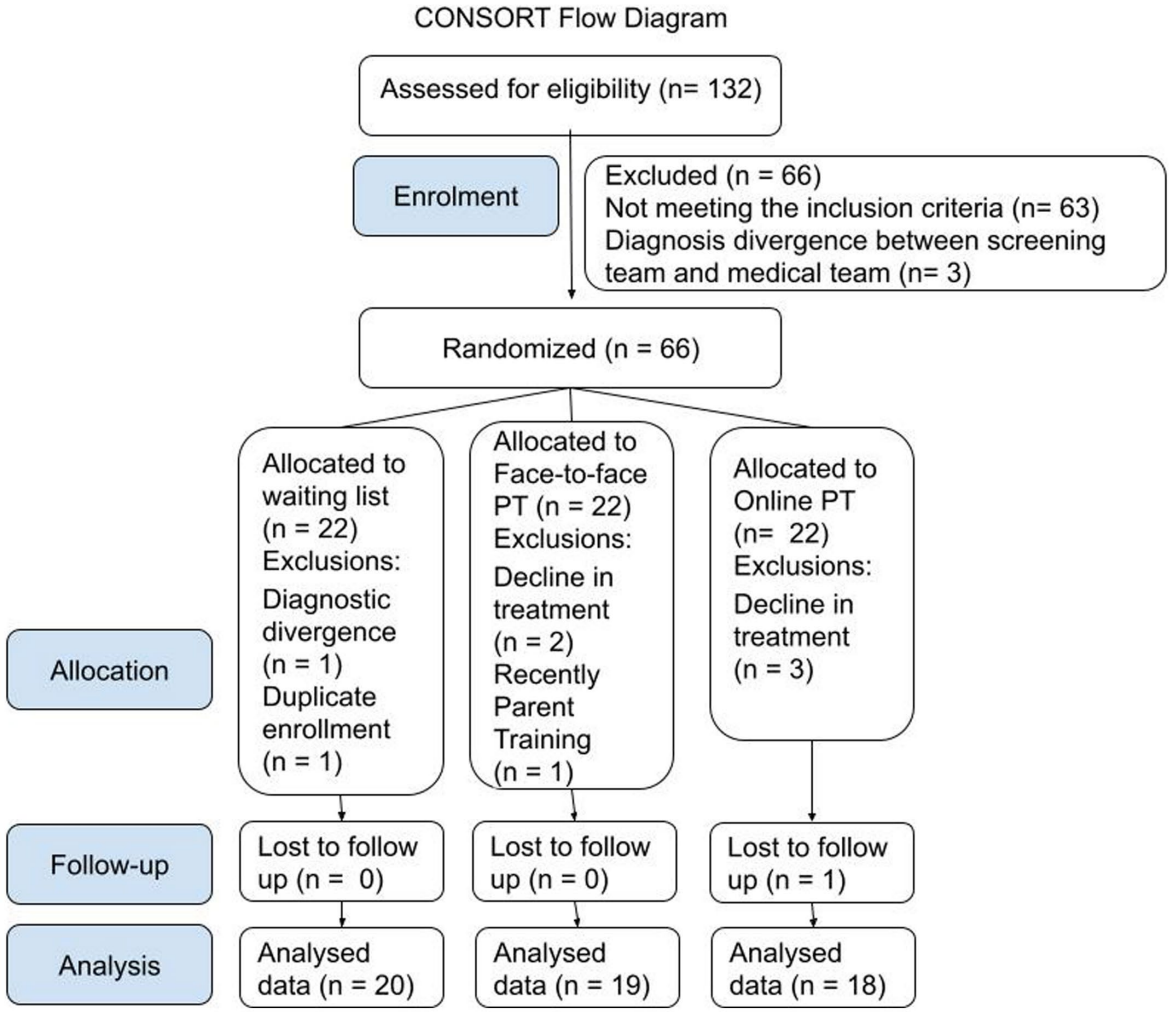
Participants enrolment, allocation, follow-up and analysis according to CONSORT guidelines.

After randomization, the participants underwent a pre-intervention assessment, filled out digitally and in person at the outpatient clinic. After that, they were immediately referred to the first consultation with the childhood and adolescent psychiatry medical team. At the beginning of the medical follow-up, if there was a divergence of diagnosis in relation to screening assessment, the team discussed the case and if necessary, the participant could be excluded, maintaining the possibility of accessing our online parent training. Soon after the pre-intervention assessment and the beginning of the medical follow-up, the allocation of the participant was revealed to the specialized therapist responsible for the interventions, who contacted the family to schedule the start of the face-to-face or online intervention. The control group waited for about 6 weeks and then was directed to the post-evaluation. Upon completion of the post-assessment, control participants had access to online parenting training. The other groups underwent the same post-assessment at the end of the interventions. For participants’ CONSORT flowchart, see Figure 1.

Parent training was planned based on Kazdin’s (2005) directions but modified for six sections. Six face-to-face sessions and six analogs online modules were developed, in which management strategies are taught to parents, with the aim of increasing caregivers’ control over children’s behavior, teaching and strengthening adaptive behaviors, and putting potentially harmful behaviors into extinction. The intervention proposed weekly appointments, but there were variations between the groups regarding the time for completion due to reschedule needs. In the online sessions/modules, the content was exposed by a short film with common examples, and at the end, support materials were made available, which always had a booklet summarizing the seen content and other materials, such as a chart of illustrations for that the caregiver can visually assemble the routine with the child.

In brief, in session/module 01, the following points are worked on: ABC of behavior; the importance of clarity in describing problem behaviors and the concept of positive opposite behavior; efficient strategies when giving commands; and routine and the importance of consistency. In session/module 02: The reinforcing consequences; the power of praise and a more enthusiastic way of delivering it that more efficiently selects behavior for having a greater magnitude; and the incentive board as a very useful and dynamic tool. In session/module 03: punitive consequences as a complement to reinforcement strategies; time out reinforcement; the strategy of ignoring bad behavior; the reprimand for behaviors that need to be stopped; and the loss of privileges. In session/module 04: modeling as a systematic teaching process, necessary for the effectiveness of interventions in many cases—adapting the level of demand is part of the proposal to create contingencies so that the appropriate behavior occurs and can be reinforced. In session/module 05: Review and punitive task for more serious and atypical behaviors. In session/module 06: Conflict resolution/negotiation + application of learned strategies to example situations.

All sessions/modules have a summary booklet, in addition to the following materials: behavior record sheet and routine illustrations kit (mod. 01); incentive board and poster for parents, showing the main strategies when giving commands (mod. 02); punishment planning sheet (mod. 03); sheet for modeling planning and modeling sheet for school (mod. 04); and cards with step-by-step and trading rules (mod. 06). The download is available upon completion of the module for all online participants and was delivered in print to face-to-face participants.

On the online platform, the intervention is 100% self-directed, with no contact with the therapist, and the videos already seen can be watched as many times as the participant needs. There is an introductory video with the main therapist explaining the intervention, and the use of the platform and an email address are available for operational questions. The content is exposed through animations, narrated by the main therapist of the project, and divided into several sequential videos of a maximum of 10 min each (the number of videos and durations may vary according to the module). Once one module is complete, the following content is only released after a week, and the day before completing a week, parents receive teasers about the content to be seen the next day.

The face-to-face intervention was carried out by psychologists from the Research Center of Impulsivity and Attention (NITIDA), previously trained and with at least 5 years of experience in behavioral psychology. For the two intervention groups, the maximum distance allowed between sessions/modules was 60 days, considering the feasibility of the project and participants being always encouraged to complete it weekly. In addition to the teasers, the technician responsible for the online platform is kept in direct contact with the online participants to remind them of deadlines and ask about technical difficulties. To ensure that the caregiver watched and absorbed the content, a quick test covering what was worked on was requested after the completion of each online module, requiring at least 60% success for participants. The duration of the sessions/modules varies, ranging from 1 h to 1 h 30 min in the face-to-face format (depending on the content, caregivers’ doubts, and dynamics of the session) and from 20 to 40 min in the online format (depending on the content).

From the pre-evaluation to the start of the intervention/waiting time and from the end of the intervention/waiting time to the post-evaluation, the maximum interval was 60 days, considering the feasibility of the project and being scheduled as soon as possible according to the availability of the patients. The post-assessment consisted of a battery of validated scales to be filled out digitally, in person at NITIDA, in addition to a new clinical interview (same conducted in screening) focusing on the symptoms of ADHD and ODD, applied by a team of specialized psychologists, blinded to the group. The entire team responsible for recruitment and evaluations was blinded to the type of intervention performed on each participant. Descriptive statistics regarding sociodemographic and clinical variables of the children and their parents are shown in Table 1.

**Table 1:** Participants’ description.

Variables			ST (*N* = 20)	ST + On-line PT (*n* = 18)	ST + Face-to-Face PT (*n* = 19)	*p*
Age (child)	In years	M ± SD	9.6 ± 1.8	8.2 ± 1.6	8.0 ± 1.6	0.014
Age (parent)	In years	M ± SD	41.1 ± 6.4	40.1 ± 4.4	39.5 ± 4.9	0.648
IQ (child)	Raven colored matrices (*Z*-score)	M ± SD	0.2 ± 0.7	0.4 ± 0.8	0.4 ± 0.9	0.720
IQ (parent)	WMT-II (*Z*-score)	M ± SD	−0.4 ± 0.8	0.2 ± 0.8	0.0 ± 1.0	0.404
Symptoms of anxiety (parent)	Sum DASS	M ± SD	6.05 ± 5.01	3.72 ± 3.08	4.72 ± 4.57	0.718
Symptoms of depression (parent)	Sum DASS	M ± SD	5.1 ± 4.42	6.16 ± 5.65	6.36 ± 5.56	0.259
Symptoms of stress (parent)	Sum DASS	M ± SD	9.35 ± 4.11	8.05 ± 5.16	9 ± 5.50	0.711
Symptoms of Inattention (parent)	ASRS number of symptoms	M ± SD	4.2 ± 3.32	2.38 ± 2.35	2.52 ± 2.59	0.090
Symptoms of Hyperactivity-Impulsivity (parent)	ASRS number of symptoms	M ± SD	3 ± 2.29	2.72 ± 2.40	1.84 ± 1.68	0.225
Duration of the intervention	In weeks	M ± SD	0.0 ± 0.0	10.8 ± 4.0	7.7 ± 1.4	
Educational level (children)	Kindergarten	*n* (%)	0 (0%)	1 (6%)	0 (0%)	0.135
	Elementary school (first year)	*n* (%)	1 (5%)	1 (6%)	4 (21%)	
	Elementary school (second year)	*n* (%)	0 (0%)	2 (11%)	4 (21%)	
	Elementary school (third year)	*n* (%)	3 (15%)	7 (39%)	5 (26%)	
	Elementary school (forth year)	*n* (%)	6 (30%)	4 (22%)	4 (21%)	
	Elementary school (fifth year)	*n* (%)	4 (20%)	2 (11%)	0 (0%)	
	Middle school (sixth year)	*n* (%)	2 (10%)	0 (0%)	1 (5%)	
	Middle school (seventh year)	*n* (%)	4 (20%)	1 (6%)	1 (5%)	
Type of school (children)	Public	*n* (%)	14 (70%)	10 (56%)	7 (37%)	0.150
	Private	*n* (%)	6 (30%)	8 (44%)	12 (63%)	
Premature birth (children)	Yes	*n* (%)	6 (30%)	2 (11%)	4 (21%)	0.403
Previous psychiatric diagnosis (children)	None	*n* (%)	3 (15%)	4 (22%)	4 (21%)	0.818
	ADHD	*n* (%)	12 (60%)	7 (39%)	9 (47%)	
	ODD	*n* (%)	0 (0%)	0 (0%)	1 (5%)	
	ADHD + ODD	*n* (%)	4 (20%)	2 (11%)	4 (21%)	
	ADHD + ASD	*n* (%)	0 (0%)	1 (6%)	0 (0%)	
	ADHD + Anxiety	*n* (%)	0 (0%)	1 (6%)	1 (5%)	
	ADHD + ODD + ASD	*n* (%)	1 (5%)	2 (11%)	0 (0%)	
	ADHD + ODD + OCD	*n* (%)	0 (0%)	1 (6%)	0 (0%)	
Prior treatment regime	None	*n* (%)	9 (45%)	9 (50%)	9 (47%)	0.954
	Psychostimulant	*n* (%)	4 (20%)	4 (22%)	1 (5%)	
	Neuroleptic	*n* (%)	3 (15%)	1 (6%)	2 (11%)	
	Antidepressants	*n* (%)	0 (0%)	0 (0%)	1 (5%)	
	Psychostimulant + Neuroleptics	*n* (%)	3 (15%)	2 (11%)	2 (11%)	
	Neuroleptics + Antidepressants	*n* (%)	0 (0%)	0 (0%)	1 (5%)	
	Psychostimulants + Antidepressants	*n* (%)	0 (0%)	0 (0%)	2 (11%)	
	Psychostimulants + Neuroleptics + Antidepressants	*n* (%)	0 (0%)	1 (6%)	0 (0%)	
	Psychostimulants + Neuroleptics + Other	*n* (%)	1 (5%)	0 (0%)	1 (5%)	
	Psychostimulants + Neuroleptics + Antidepressants + Others	*n* (%)	0 (0%)	1 (6%)	0 (0%)	
Prior non-pharmacological interventions	Speech therapist (prior)	*n* (%)	7 (35%)	10 (56%)	11 (58%)	0.290
	Psychotherapy (prior)	*n* (%)	19 (95%)	13 (72%)	17 (89%)	0.11
	Occupational therapy (prior)	*n* (%)	2 (10%)	4 (22%)	4 (21%)	0.543
	Educational psychology (prior)	*n* (%)	4 (21%)	5 (29%)	6 (32%)	0.745
Medications started concomitantly with PT	None	*n* (%)	13 (65%)	13 (72%)	14 (74%)	0.836
	Psychostimulant	*n* (%)	3 (15%)	5 (28%)	5 (26%)	
	Antidepressant	*n* (%)	2 (10%)	0 (0%)	0 (0%)	
	Psychostimulants + Neuroleptics	*n* (%)	1 (5%)	0 (0%)	0 (0%)	
	Psychostimulants + Antidepressants	*n* (%)	1 (5%)	0 (0%)	0 (0%)	

WMT-II, Viena Matrices Test 2; DASS, Depression, Anxiety, and Stress Scale; ADHD, Attention Deficit Hyperactivity Disorder; ODD, Oppostive-Defiant Disorder; ASD, Autism Spectrum Disorder; OCD, Onsessive Compulsive Disorder.

### Measures

For the *screening process* and semi-structured diagnostic investigation applied by a trained professional, the Kiddie Schedule for Affective Disorders and Schizophrenia for School Aged Children—Lifetime Version (K-SADS-PL; clinical interview) was used, which is based on the DSM-5 criteria (American Psychiatric Association, 2014). The interview investigated all symptoms described in the Diagnostic and Statistical Manual of Mental Disorders (DSM-5) and was applied to the primary caregiver by a trained psychologist and a member of NITIDA. For the assessment of the caregiver’s and the child’s intelligence, the Vienna (Waldherr et al., 2014) and Raven matrices tests (Pasquali et al., 2002) (respectively) were used, being both validated and standardized in the Brazilian context, and are intended to assess fluid intelligence or logical reasoning.

The *pre-intervention assessment* had a battery of scales and questionnaires to be filled in by the caregiver, some of which were self-reported and others related to the child’s behavior, in addition to a standardized socioeconomic status questionnaire [Associação Brasileira de Empresas de Pesquisa (ABEP), 2015]. All the procedures were done according to the previously published protocol (Chequer de Castro Paiva et al., 2022). Caregivers filled out the following scales:

Adult Self-Report Scale (ASRS-18) (Leite, 2011): 18-item self-report measure of ADHD symptoms in adults. Scores range from 0 to 36 for the subscales Inattention and Hyperactivity-Impulsivity. Higher scores indicate more symptoms. Reliability for the Brazilian version, according to Leite (2011), is 0.938.

Multimodal Treatment Study version of the Swanson, Nolan, and Pelham ADHD scale version IV (MTA-SNAP-IV) (Costa et al., 2019): a 26-item parent-report measure of ADHD and ODD symptoms in children. Scores Range from 0 to 27 for the Inattention and Hyperactivity-Impulsivity subscales and 0 to 24 for the ODD subscale. Higher scores indicate more symptoms. Reliability for the Brazilian version, according to Costa et al. (2019), varies from 0.92 to 0.94 depending on the method and subscale.

Depression, Anxiety, and Stress Scale (DASS) (Vignola, and Tucci, 2014): an instrument designed to assess specific symptoms of depression, anxiety, and stress. Scores range from 0 to 21 for each subscale. Higher scores indicate more symptoms. Reliability for the Brazilian version ranges from 0.86 to 0.92, according to the subscale.

Perceived Stress Scale (PSS) (Faro, 2015): A standardized measure of perceived stress using positive and negative questions. We used the 14-item version in this study. Scores range from 0 to 56, and scores indicate higher perceived stress and reliability for the Brazilian version, according to Faro (2015), is 0.77.

Parenting Styles and Dimensions Questionnaire (PSDQ) (Oliveira et al., 2018): A self-reported questionnaire designed to assess parental behavioral/educational methods. It has multiple subscales representing three main facets of parental styles—authoritative, authoritarian, and permissive. Scores for each dimension range from 1 to 5. Higher scores indicate more usage of a specific style. Reliability for the Brazilian version, according to Oliveira et al. (2018), is 0.775.

World Health Organization Quality of Life abbreviated measure (WHOQOL-BREF; WHO) (Fleck et al., 2000): An instrument designed to assess four border domains of quality of life: physical, social, psychological, and environmental. Standardized scores range from 0 to 100. Higher scores indicate higher quality of life. Reliability for the Brazilian version, according to Fleck and colleagues, ranges from 0.69 to 0.91.

The Child and Adolescent Behavior Inventory (CABI) (Cianchetti et al., 2017): the CABI is a multidimensional inventory to assess different areas of children's behavior and psychopathology. Its main scores compute externalizing symptoms (ODD, conduct disorder, emotional instability…) and internalizing symptoms (depression, anxiety, somatic problems…), as well as ADHD symptoms. Scores range from 0 to 28 (Internalizing), 0 to 20 (Externalizing), and 0 to 18 (ADHD). Higher scores are indicative of more symptoms. Reliability, according to Costa et al. (2023), ranges from 0.88 to 0.91, according to subscale and method.

Kidscreen-52 (Guedes and Guedes, 2011): It is a 5-point Likert scale, varying excellent–bad; nothing–extremely; and never–always. The questionnaire investigates the quality of life in children in 10 dimensions: physical wellbeing; psychological wellbeing; moods and emotions; self-perception; autonomy; parent relation and home life; financial resources; social support and peers; school environment; and social acceptance (bullying). After correction (considering inverse items), higher scores denote higher quality of life in each domain. Reliability, according to Guedes and Guedes (2011), ranges from 0.72 to 0.88 according to dimensions.

The questionnaires related to the expected outcomes in this trial and reapplied in the *post-intervention evaluation* were MTA-SNAP-V, PSSP, SDQ, WHOQOL-BREF, and Kidscreen.

### Randomization

Participants were randomly assigned to either control or one of the experimental groups with a 1:1 allocation as per a computer-generated randomization schedule. The allocation sequence was generated applying a permuted block design with random blocks stratified by children’s age. All patients who gave consent forms and who fulfilled the inclusion criteria were randomized. Randomization was requested by the staff member responsible for recruitment and clinical interviews and was performed by the computer technician, both members of NITIDA. Closed envelopes with printed randomized numbers on them were available for the therapist, who was not involved in assessing the outcome of the study. Once the initial assessment was completed, the therapist verified the allocation, and the intervention was initiated. Staff responsible for recruitment and symptom ratings were not allowed to receive information about the group allocation.

### Statistical analysis

The researchers responsible for data management and statistical analysis were blind to the research groups. Baseline comparisons were performed using the chi-square test for categorical data and one-way ANOVA for dimensional data. Our sample size offers 99% power to detect large (0.40) or moderate effect sizes (0.25) and 82% for small (0.15) effect sizes with a 5% error probability. We estimated the correlation between repeated measures using the test–retest reliability coefficient for the psychometric tests (an average of 0.7). The power analysis was conducted in the G*Power 3.1.9.7 software (Faul et al., 2007).

The comparison between pre- and post-intervention measures was performed using analysis of variance for repeated measures. Our sample had missing data post-intervention. Although there was little missing data, the sample available for each variable varied between 54 and 57, with an average of 56.4. A visual analysis of missing data and the MCAR test suggested a pattern in which the variables were missing completely at random, so we used multiple imputations using the automatic method (based on linear regression) of SPSS 25.0 (IBM Corp. Released, 2017). All available sociodemographic, baseline, and outcome measures were used as predictors. We computed 10 virtual datasets using this method and condensed them into a unique database for further analysis, containing the mean of each variable across the 10 imputations for each subject.

We specifically analyzed the effect of the interaction between the time factor (pre × post-intervention) and the group factor (ST × ST + Online PT × ST + Face-to-Face PT) in terms of statistical significance and effect size (using the *partial eta-square*). Age was added as a covariate. For this calculation, we typically considered a small effect size of 0.01, moderate values of 0.06, and large values of 0.15 (Hair et al., 2009). Post-hoc analyses were performed using the Bonferroni–Holm method for multiple comparisons. The latter procedures were performed in JASP 0.16.4 (JASP Team, 2022).

## Results

### Effects of the interventions on ADHD symptoms

**Figure 2:**
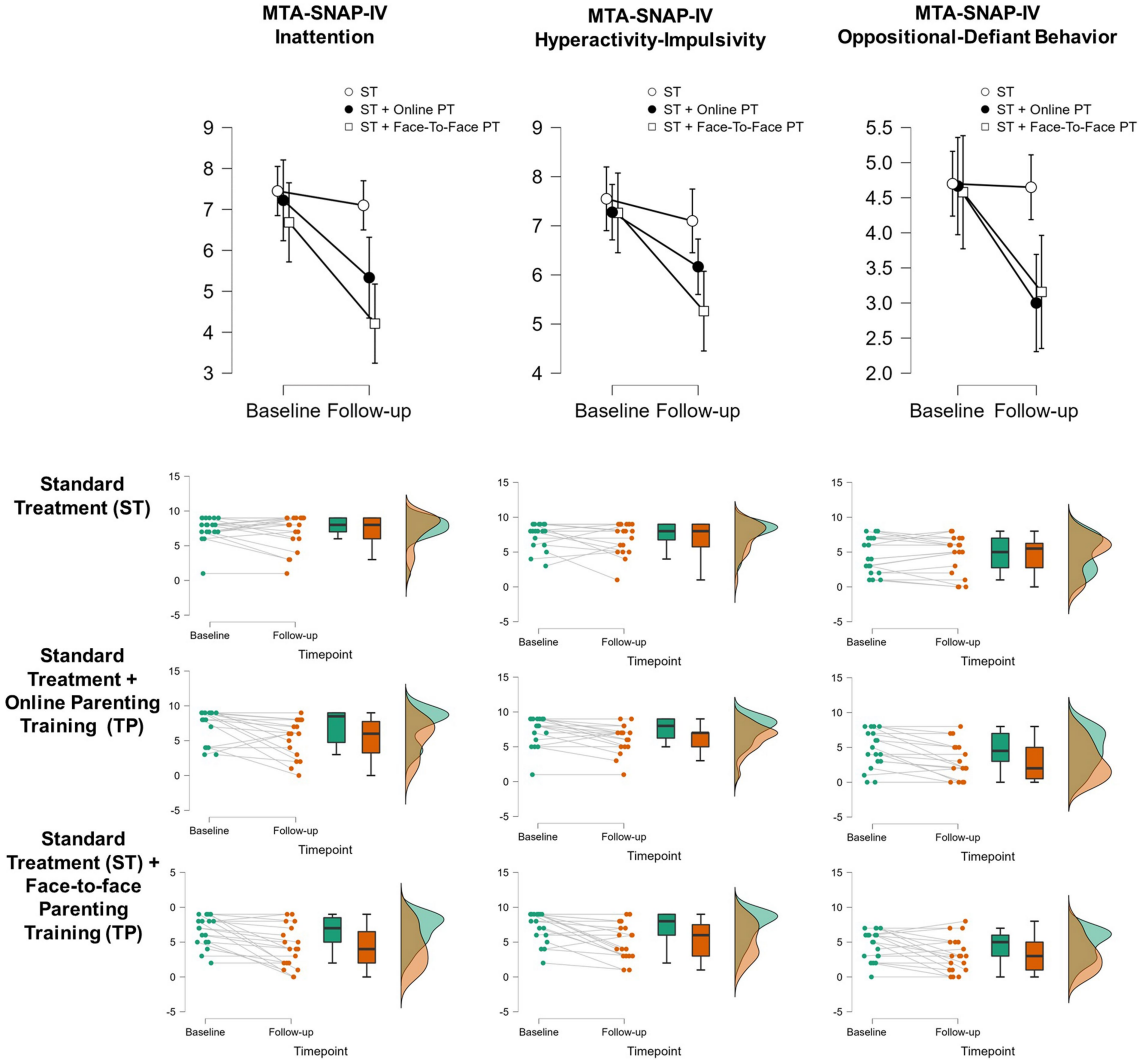
Comparisons of ADHD symptoms pre and post-treatment, stratified by group (repeated measures ANOVA). The upper panel shows the mean MTA-SNAP-IV scores for each subscale between baseline and follow-up assessments, while the following panels report individual and group data for each comparison. All treatment groups showed a significant reduction in ADHD symptoms between the two time points (*p* < 0.001). We found interactions between treatment modality and time-point for symptoms of inattention (*p* < 0.030) and oppositional-defiant behavior (*p* = 0.026). For inattention, Standard Treatment (ST) in addition to face-to-face parent training (PT) showed a significant improvement between the two assessments (*p* < 0.001) when compared to ST alone. For oppositional-defiant behavior, the addition of face-to-face PT (*p* = 0.033) or online PT (*p* = 0.009) was associated with a significant reduction in symptoms, while ST alone wasn’t (*p* = 1.000).

The results regarding the effect of the interventions on ADHD symptoms can be found in Figure 2. The comparisons between pre- and post-treatment were significant for all MTA-SNAP-IV measures, reflecting an important symptom reduction: inattention (*F* (54) =22.23, *p* < 0.001, η_p_^2^ = 0.29), hyperactivity-impulsivity (*F* (54) =19.79, *p* < 0.001, η_p_^2^ = 0.27), and ODD (*F* (54) =16.51, *p* < 0.001, η_p_^2^ = 0.13).

Regarding treatment outcomes, we found a significant interaction between timepoint (baseline × follow-up) and treatment modality (ST, ST + Online PT, ST + Face-to-Face PT) for MTA-SNAP-IV inattention (*F* (54) =3.72, *p* = 0.030, η_p_^2^ = 0.12) and MTA-Snap-IV ODD (*F* (54) =3.89, *p* = 0.026, η_p_^2^ = 0.13) but not for MTA-SNAP-IV hyperactivity (*F* (54) =2.90, *p* = 0.063). These results are summarized in Figure 2.

There were no group differences in baseline. Post-hoc analysis (corrected for multiple comparisons using the Bonferroni–Holm method) for MTA-SNAP-IV inattention suggests that ST alone did not show significant improvement between pre- and post-treatments (mean difference = 0.35, *p* = 1.000), but the addition of Face-to-Face PT does (mean difference = 3.24, *p* = 0.001). ST + Online TP showed a similar trend but did not reach statistical significance (mean difference = 2.11, *p* = 0.093). The comparison between the PT modalities showed no significant differences (mean difference = 1.12, *p* = 1.000).

For MTA-SNAP-IV ODD symptoms, ST alone also did not reduce symptoms between baseline and follow-up (mean difference = 0.050, *p* = 1.000), but the addition of Face-to-Face PT (mean difference = 1.49, *p* = 0.033) or online PT (mean difference = 1.65, *p* = 0.009) does it. There was no significant difference between PT modalities (mean difference = −0.16, *p* = 1.000).

### Effects of the interventions on parental styles

Table 2 presents the results regarding the PSDQ measures. In addition to the subscales, support and affection (*p* = 0.081) showed significant differences between pre- and post-treatment [average effect size (*η*_p_^2^ = 0.13)]. These differences were all toward positive parenting (reduction of authoritarian and permissive methods and increase of authoritative ones). We found a significant interaction between pre-/post-intervention and treatment modality in PSDQ support and affection subscales—a component of the Authoritative Parental Style (*p* = 0.005), but post-hoc analyses were not significant after correcting multiple comparisons. The other results of PSDQ main scores or subscales did not reach statistical significance.

**Table 2:** Comparison between parents styles and dimensions questionnaire (PSDQ) scores pre- and post-treatment and interaction with treatment modality.

PSDQ dimensions	Group	Pre-treatment	Post-treatment	Time			Time × Group		
				*F*(df)	*p*	*Post hoc*	*F*(df)	*p* (*n*_p_^2^)	*Post hoc*
Suport and affection	ST	4.21 ± 0.57	4.47 ± 0.57	1.00 (53)	0.320	–	6.22 (53)	0.004	NS
	ST + Online PT	4.08 ± 0.54	4.60 ± 0.35					(*n*_p_^2^ = 0.19)	
	ST + Face-to-Face PT	4.30 ± 0.38	4.78 ± 0.74						
Regulation	ST	4.23 ± 0.55	4.68 ± 0.45	3.59 (52)	0.064	–	1.95 (52)	0.028	NS
	ST + Online PT	4.21 ± 0.73	4.62 ± 0.43					(*n*_p_^2^ = 0.13)	
	ST + Face-to-Face PT	4.53 ± 0.47	4.49 ± 0.66						
Autonomy	ST	3.55 ± 0.51	3.89 ± 0.56	0.53 (53)	0.468	–	1.85 (53)	0.168	–
	ST + Online PT	3.32 ± 0.82	3.92 ± 0.48						
	ST + Face-to-Face PT	3.60 ± 0.56	3.61 ± 0.61						
Physical coercion	ST	2.37 ± 0.98	1.81 ± 0.66	0.01 (53)	0.493	–	0.48 (53)	0.954	–
	ST + Online PT	2.33 ± 0.71	1.86 ± 0.82						
	ST + Face-to-Face PT	2.00 ± 0.85	1.53 ± 0.60						
Verbal Hostility	ST	2.88 ± 0.75	2.72 ± 0.94	1.59 (53)	0.213	–	0.153 (53)	0.858	–
	ST + Online PT	2.88 ± 0.83	2.38 ± 0.54						
	ST + Face-to-Face PT	2.69 ± 0.72	2.14 ± 0.73						
Punishment	ST	2.15 ± 0.60	1.85 ± 0.58	1.73 (53)	0.194	–	0.57 (53)	0.571	–
	ST + Online PT	2.16 ± 0.61	1.65 ± 0.81						
	ST + Face-to-Face PT	2.07 ± 0.54	1.85 ± 0.58						
Authoritative style	ST	3.97 ± 0.30	4.23 ± 0.45	3.83 (53)	0.089	–	3.84 (53)	0.028	NS
	ST + Online PT	3.89 ± 0.48	4.28 ± 0.25					(*n*_p_^2^ = 0.13)	
	ST + Face-to-Face PT	4.30 ± 0.28	4.32 ± 0.49						
Authoritarian style	ST	2.47 ± 0.63	2.12 ± 0.56	1.56 (53)	0.214	–	0.05 (53)	0.951	–
	ST + Online PT	2.46 ± 0.54	1.96 ± 0.79						
	ST + Face-to-Face PT	2.25 ± 0.55	1.84 ± 0.56						
Permissive style	ST	2.42 ± 0.44	2.04 ± 0.43	0.01 (53)	0.966		0.90 (53)	0.415	–
	ST + Online PT	2.32 ± 0.46	2.14 ± 0.54						
	ST + Face-to-Face PT	2.19 ± 0.28	1.97 ± 0.37						

ST, Standard Treatment; PT, Parent Training; NS, non-significant after multiple-comparisons correction (Bonferroni method).

### Children’s quality of life

The results of this section are shown in Table 3. Our analysis suggests a significant improvement in the psychological, mood and emotion, parents, financial, and school aspects of the patient’s quality of life in the Kidscreen-52 (al *p* < 0.05, average effect size of of η_p_^2^ = 0.13) but non-significant results for physical, self-perception, autonomy, or peers. We found an interaction between pre−/post-treatment and treatment modality only in the physical domain of quality of life (*F* (54) =5.22, *p* = 0.009, η_p_^2^ = 0.16). Post-hoc analysis suggests improved quality of life when the ST was compared to both in ST + Online PT (mean difference = −5.90) and ST + the Face-to-Face PT (mean difference = −5.25), although these differences did not remain after adjusting for multiple comparisons (*p* = 0.444 and *p* = 0.556, respectively).

**Table 3:** Comparison between children’s quality of life in the Kidscreen-52 questionnaire reported by their parents’ pre- and post-treatments and interaction with treatment modality.

Kidscreen-52	Group	Pre-treatment	Post-treatment	Time			Time × Group		
				*F*(df)	*p*	*Post hoc*	*F*(df)	*p* (*n*_p_^2^)	*Post hoc*
Physical	ST	46.31 ± 10.10	43.98 ± 11.00	3.64 (53)	0.062	Post > Pre	5.22 (54)	0.009 (*n*_p_^2^ = 0.16)	ST < Online PT
	ST + Online PT	45.64 ± 9.90	49.88 ± 9.13						ST < Face to
	ST + Face-to-Face PT	46.44 ± 8.42	49.77 ± 9.23						Face PT
Psychological	ST	45.22 ± 11.22	45.38 ± 10.55	7.00 (52)	0.011	Post > Pre	1.70 (52)	0.192	–
Wellbeing	ST + Online PT	45.38 ± 10.53	49.80 ± 11.04		(*n*_p_^2^ = 0.12)				
	ST + Face-to-Face PT	46.49 ± 10.46	52.38 ± 11.32						
Moods and emotion	ST	14.95 ± 7.68	12.90 ± 9.07	10.35 (54)	0.002	Post > Pre	0.01 (54)	0.889	–
	ST + Online PT	12.08 ± 9.24	12.16 ± 6.03		(*n*_p_^2^ = 0.16)				
	ST + Face-to-Face PT	12.71 ± 8.34	9.67 ± 8.10						
Self-perception	ST	36.07 ± 5.85	34.18 ± 4.47	0.01	0.922	–	2.55	0.087	–
	ST + Online PT	33.75 ± 4.06	35.24 ± 5.71						
	ST + Face-to-Face PT	33.50 ± 4.81	34.06 ± 2.44						
Autonomy	ST	47.84 ± 8.90	47.56 ± 8.90	0.65 (51)	0.424	–	0.86 (51)	0.430	–
	ST + Online PT	44.08 ± 11.14	44.08 ± 11.14						
	ST + Face-to-Face PT	44.06 ± 9.01	44.07 ± 9.01						
Parents	ST	41.00. ± 10.84	43.21 ± 11.11	5.24 (54)	0.026	Post > Pre	0.33 (54)	0.716	–
	ST + Online PT	39.09 ± 8.17	42.13 ± 7.45		(*n*_p_^2^ = 0.09)				
	ST + Face-to-Face PT	40.55 ± 9.20	45.06 ± 11.40						
Financial	ST	42.20 ± 8.79	43.84 ± 11.39	7.08 (53)	0.010	Post > Pre	0.70 (53)	0.501	–
	ST + Online PT	39.20 ± 10.93	45.06 ± 9.13		(*n*_p_^2^ = 0.12)				
	ST + Face-to-Face PT	43.24 ± 12.00	47.40 ± 10.33						
Peers	ST	44.96 ± 10.87	45.45 ± 12.30	2.37 (54)	0.131	–	0.46 (54)	0.635	–
	ST + Online PT	37.43 ± 14.30	42.06 ± 14.11						
	ST + Face-to-Face PT	42.56 ± 13.13	45.78 ± 12.35						
School	ST	42.87 ± 9.10	43.48 ± 8.61	11.35 (52)	0.001	Post > Pre	2.17 (52)	0.125	–
	ST + Online PT	42.55 ± 12.84	47.97 ± 8.99		(*n*_p_^2^ = 0.18)				
	ST + Face-to-Face PT	42.95 ± 11.15	49.26 ± 9.53						

ST, Standard Treatment; PT, Parent Training; NS, non-significant after multiple-comparisons correction (Bonferroni method).

### Parent’s quality of life and perceived stress

The results of this section are shown in Supplementary Table S1. Quality of life in the physical (*F* (47) =7.54, *p* = 0.009, η_p_^2^ = 0.14), psychological (*F* (52) =8.59, *p* = 0.005, η_p_^2^ = 0.14), and environmental domains (*F* (49) =8.93, *p* = 0.004, η_p_^2^ = 0.15) were higher at the post-treatment assessment when compared to pretreatment. No differences were observed in the social domain of quality of life or in the perceived stress scale. No interactions with treatment modality were significant in these analyses.

## Discussion

The study findings suggest that response to intervention of symptoms of inattention and oppositional defiant behaviors differs among groups. There was a greater improvement in the PT group, with the face-to-face and online PT in relation to symptoms of inattention and ODD. Regarding inattention, the confidence interval bars in the post-test do not overlap in the face-to-face and control groups, indicating a significant difference between the means of these groups. For ODD symptoms, there is a very similar pattern of symptom decline in the intervention groups and stability of symptoms in the group on the waiting list.

Interestingly, a recent randomized clinical trial by Hornstra et al. (2021) identified characteristics of parent training that would be related to the reduction of ADHD symptoms. The three-arm study had a control group and two intervention groups; one focused on antecedent strategies or stimulus control, and the other focused on consequences or contingency management. Research identified a reduction in children’s behavior problems and hyperactivity/impulsivity symptoms in both interventions, with no significant changes in ODD symptoms. In the background-focused intervention group, the decrease in inattention symptoms was significantly more significant compared to the other groups. In a way, our results confirm the potential of psychosocial interventions to reduce ADHD symptoms (Hornstra et al., 2021).

However, many review studies question the effectiveness of parent training in reducing ADHD symptoms, highlighting a more significant relationship between the intervention and functional outcomes (Evans et al., 2014; Daley et al., 2018; Caye et al., 2019; Drechsler et al., 2020). Limitations related to information blinding are a reality of studies and should be a matter of concern (Caye et al., 2019; Rimestad et al., 2019). It is worth noting that a change in parents’ perception of the child’s symptoms can also be a positive outcome, as it may be related to improved quality of life and parental stress (Trivedi, 2017). In addition, more specific aspects related to the content of programs can shed light on what underlies the observed effects (Hornstra et al., 2021; Dekkers et al., 2022). Beyond the strength of testing an intervention in a different scenario, there are two clear limitations of this study: it refers to the findings’ generalization and about reliability of the treatment. Since this study was done in a small sample with specific characteristics, it may fail in the generalization of the findings for middle-income populations. The intervention was adapted from the model proposed by Kazdin (2005) by a team of psychologists, and the learned content was measured, but no objective measure of fidelity and satisfaction with the treatment was obtained.

Regarding ODD symptoms, parent training is effective and is the first-choice treatment (Do Austerman, 2015; Caye et al., 2019; Kaur et al., 2022). Irritability and defiance, two dimensions of ODD, might have a heterogeneous response to interventions (Zachary and Jones, 2019). In this clinical trial, only the number of ODD symptoms was reported. There is a need to develop more specific and effective treatments with larger samples, multiple measurements, and multivariate analytical approaches (Posner et al., 2020).

In terms of secondary outcomes, the results showed significant differences regarding support and affection parenting dimensions and marginally significant differences in relation to democratic parenting style. The graph indicates the stability of democratic strategies in the face-to-face group, and there is a significant improvement in the online group in relation to the face-to-face group. Support and affection dimensions present similar responses online and face-to-face. It highlights the potential for the online platform to improve aspects of parenting compared to face-to-face interventions.

Negative parenting, authoritarianism, excess control through punitive strategies, and less parental responsiveness toward their children result in the worsening of children’s externalizing problems (Modesto-Lowe et al., 2008; Pinquart, 2017). Changing parenting behavior is, therefore, an obvious way to break this cycle. The democratic style balances the affection and control dimensions, which can be interpreted as positive parenting (Oliveira et al., 2018). Positive parenting and reduced parental stress are effects reported regarding PT and are related to children’s behavioral change (Dekkers et al., 2022). Parental training seeks to change parental behavior and, therefore, changes in children’s behavior mediated by parental behavior. Investigating the mediation relationships between parenting and specific children’s behavioral changes can be important to understanding the mechanisms underlying the intervention and potentially improving therapeutic planning (Forehand et al., 2014). Despite being a consistent result in literature, no significant differences were observed related to caregivers’ perceived stress.

Regarding parents’ quality of life, results showed significant differences for physical health. The confidence interval bars of the means overlap for the three groups in the pre- and post-test, but the visual analysis indicates a pattern of stability in the control group and improvement in the intervention groups in physical wellbeing and self-perception of children, without differences in the means in the pre- or post-test. Regarding social acceptance, the differences in the pre-test were significant for children in the control and online intervention groups, indicating an improvement in the online group in relation to the usual treatment, with overlapping confidence interval bars of the post-test means. Online intervention seems to be effective for the stability or improvement of aspects related to the social acceptance of children, which may be related to the reduction of social impairments.

A previous meta-analysis demonstrated a negative impact of ADHD on physical and psychosocial quality of life, with moderate and large effect sizes, respectively (Lee et al., 2016). In a study published in 2021, Larsen and colleagues observed through a clinical trial that children with ADHD negatively impact health-related quality of life and that parent training has the potential to improve this impairment, regardless of the effects on symptoms (Larsen et al., 2021).

Online interventions have been proposed and validated, including parent training (Baumel et al., 2016, 2017; Thongseiratch et al., 2020). The effects seem like face-to-face intervention and are especially important when considering unassisted children (Baumel et al., 2017). Considering these findings, it is reasonable to think about online parent training as a good low-cost possibility for poor and middle-income contexts. However, given the nature of the online format, utilizing the platform is most effective when preceded by a confirmed diagnosis, ensuring a precise and tailored intervention.

During the pandemic, data collection faced some limitations in sample size. However, it is crucial to explore the impact of large-scale public psychosocial interventions specifically tailored for children with ADHD and their families. This exploration allows us to explore how different contexts influence the response to these interventions. While a few efforts have delved into the effectiveness of interventions involving caregivers (Russo et al., 2021), these initiatives often rely on trained professionals, incurring significant costs compared to self-directed approaches. It is worth noting that a recent Brazilian study showed an association between low socioeconomic status and negative parenting practices (Altafim et al., 2018), highlighting the importance of initiatives that broaden access to effective interventions such as parent training, particularly in enhancing parental strategies.

Future research should map aspects of the heterogeneity of the clinical response, such as (1) different health professionals’ training; (2) different ages and their responses; and (3) maintenance of strategies after interventions for families who had access to them. In addition to the PT platform being an effective intervention option, Jones et al. (2021) highlight the potential of the results of parental training when the intervention is technology-enhanced. Difficulties in maintaining improvements are evident in follow-up assessments of parent training (Trivedi, 2017). A highlighted challenge is the difficulty of engaging participants in fully self-directed models (Baumel et al., 2017; Brager et al., 2021). Reminders that could engage parents in online interventions have been pointed out as associated with effectiveness (Thongseiratch et al., 2020). Here, direct communication with the patient through email and *WhatsApp* was used to engage. Teaser emails of the next content were sent 1 day before the module was scheduled to run. Even using these strategies, the average duration of the online intervention was larger than the traditional one. Usability testing is necessary for initiatives to adapt parent training to technological models, especially considering low-income parents (Brager et al., 2021). Access to the internet must be accounted for to succeed in reaching populations.

In conclusion, parent training was effective in reducing ADHD and ODD symptoms, improving positive parenting and some aspects of children’s and parents’ quality of life. Parenting style and child’s quality of life, especially social acceptance, might also be impacted by parental training.

## Data availability statement

The raw data supporting the conclusions of this article will be made available by the authors, without undue reservation.

## Ethics statement

The studies involving humans were approved by Comitê de Ética em Pesquisa da Universidade Federal de Minas Gerais. The studies were conducted in accordance with the local legislation and institutional requirements. Written informed consent for participation in this study was provided by the participants’ legal guardians/next of kin.

## Author contributions

GC: Conceptualization, Data curation, Investigation, Methodology, Writing – original draft, Writing – review & editing. JP: Data curation, Formal analysis, Methodology, Writing – original draft, Writing – review & editing. DS: Conceptualization, Data curation, Formal analysis, Investigation, Methodology, Supervision, Writing – review & editing. AA-S: Conceptualization, Data curation, Investigation, Writing – original draft, Writing – review & editing. DF: Conceptualization, Methodology, Software, Visualization, Writing – original draft, Writing – review & editing. JJ: Investigation, Writing – original draft, Writing – review & editing. MR-S: Conceptualization, Formal analysis, Funding acquisition, Investigation, Project administration, Resources, Supervision, Writing – review & editing. DM: Conceptualization, Formal analysis, Funding acquisition, Investigation, Project administration, Resources, Supervision, Writing – original draft, Writing – review & editing.
