# Supplementary material for: Parent training for disruptive behavior symptoms in attention deficit hyperactivity disorder: a randomized clinical trial
Source: Front Psychol. 2024 Feb 16;15:1293244. doi: 10.3389/fpsyg.2024.1293244 (PMC10906662; doi:10.3389/fpsyg.2024.1293244)
Supplement: Supplementary file 1 [file Table_1.docx]

Supplementary Table 1: Comparison between parent’s quality of life and perceived stress pre and post-treatment child treatment and its interaction with treatment modality

| Measure | Group | Pre-  treatment | Post-  Treatment | Time | | |  | Time x Group | | |
| --- | --- | --- | --- | --- | --- | --- | --- | --- | --- | --- |
|  |  |  |  | F(df) | p | Post hoc |  | F(df) | p (n_p_^2^) | Post hoc |
| WHOQOL-Bref | ST | 60.51±15.76 | 58.92±16.08 | 0.15 | 0.704 | - |  | 0,12 | 0.734 | - |
| Physical | ST + Online PT | 60.71±13.86 | 70.75±15.44 | (53) |  |  |  | (53) |  |  |
|  | ST + Face-To-Face PT | 62.72±22.32 | 72.54±14.91 |  |  |  |  |  |  |  |
| WHOQOL-Bref | ST | 59.37±11.30 | 59.58±15.48 | 0.20 | 0.655 | - |  | 0.112 | 0.740 | - |
| Psychological | ST + Online PT | 54.90±14.82 | 64.23±18.34 | (53) |  |  |  | (53) |  |  |
|  | ST + Face-To-Face PT | 58.79±18.29 | 66.67±16.91 |  |  |  |  |  |  |  |
| WHOQOL-Bref | ST | 55.00±21.53 | 55.00±26.54 | 1.47 | 0.289 | - |  | 0.573 | 0.452 | - |
| Social | ST + Online PT | 45.57±26.85 | 54.90±24.66 | (53) |  |  |  | (53) |  |  |
|  | ST + Face-To-Face PT | 56.14±24.66 | 59.64±22.78 |  |  |  |  |  |  |  |
| WHOQOL-Bref | ST | 52.57±16.87 | 52.75±15.46 | 0.12 | 0.730 | - |  | 0.05 | 0.828 | - |
| Enviromental | ST + Online PT | 59.74±13.02 | 64.71±15.42 | (53) |  |  |  | (53) |  |  |
|  | ST + Face-To-Face PT | 58.51±18.59 | 67.19±15.06 |  |  |  |  |  |  |  |
| PSS | ST | 31.05±5.55 | 30.45±0.94 | 0.59 | 0.444 | - |  | 0.38 | 0.541 | - |
|  | ST + Online PT | 31.61±5.23 | 31.16±4.70 | (53) |  |  |  | (53) |  |  |
|  | ST + Face-To-Face PT | 31.63±4.54 | 30.84±4.90 |  |  |  |  |  |  |  |

ST: Standard Treatment, PT: Parent Training, NS: non-significant after multiple-comparisons correction (Bonferroni method)
